# A time-series approach to mapping livestock density using household survey data

**DOI:** 10.1038/s41598-022-16118-1

**Published:** 2022-08-03

**Authors:** Julianne Meisner, Agapitus Kato, Marshall Lemerani, Erick Mwamba Miaka, Acaga Taban Ismail, Jonathan Wakefield, Ali Rowhani-Rahbar, David Pigott, Jonathan Mayer, Peter Rabinowitz

**Affiliations:** 1 Department of Environmental and Occupational Health Sciences, Center for One Health Research, University of Washington, Seattle, 98195 USA; 2 Department of Epidemiology, University of Washington, Seattle, 98195 USA; 3 Uganda Virus Research Institute, Entebbe, Uganda; 4 Ministry of Health, Lilongwe, Malawi; 5 Programme National de Lutte contre la Trypanosomiase Humaine Africaine, Kinshasa, Democratic Republic of Congo; 6 IntraHealth International, Juba, South Sudan; 7 Department of Biostatistics, University of Washington, Seattle, 98195 USA; 8 Department of Statistics, University of Washington, Seattle, 98195 USA; 9 Department of Health Metrics Sciences, University of Washington, Seattle, 98195 USA; 10 Department of Global Health, University of Washington, Seattle, 98195 USA

**Keywords:** Epidemiology, Environmental impact, Sustainability

## Abstract

More than one billion people rely on livestock for income, nutrition, and social cohesion, however livestock keeping can facilitate disease transmission and contribute to climate change. While data on the distribution of livestock have broad utility across a range of applications, efforts to map the distribution of livestock on a large scale are limited to the Gridded Livestock of the World (GLW) project. We present a complimentary effort to map the distribution of cattle and pigs in Malawi, Uganda, Democratic Republic of Congo, and South Sudan. In contrast to GLW, which uses dasymmetric modeling applied to census data to produce time-stratified estimates of livestock counts and spatial density, our work uses complex survey data and distinct modeling methods to generate a time-series of livestock distribution, defining livestock density as the ratio of animals to humans. In addition to favorable cross-validation results and general agreement with national density estimates derived from external data on national human and livestock populations, our results demonstrate extremely good agreement with GLW-3 estimates, supporting the validity of both efforts. Our results furthermore offer a high-resolution time series result and employ a definition of density which is particularly well-suited to the study of livestock-origin zoonoses.

## Introduction

Globally, one billion people living on less than US$2 per day depend on livestock. For these communities, which include 80% of the poor in Africa, livestock are a critical source of household income, transport, draft power for crop agriculture, and nutrition, providing 11% of energy and 26% of dietary protein among the poor in East Africa, and up to 50% of energy for children under five in pastoralist communities^1,2^. Livestock keeping also plays an important cultural role, serving as a means to maintain family cohesion and social networks, gain political prestige, and strengthen legal claims on pasture land^3,4^. These critical roles livestock play, however, commonly result in close human-animal contact, driving transmission of zoonotic diseases and resulting in over 2.5 billion cases of human illness and 2.7 million deaths per year^2^.

Livestock also have major environmental impacts across a range of scales. In Africa, where an estimated 1 billion or more of the projected increase in the human population will occur, urbanization and an increased demand for animal products are also expected^5^, driving the so-called “livestock revolution^6^.” Production systems are generally low-input in sub-Saharan Africa, with very little technological change in the past 40 years^5^; compounded by degraded natural resources, this lack of technological change requires increasing production demands to be met by overgrazing and land-use changes, rather than intensification, compromising biodiversity and ecosystem services^7^. Furthermore, where intensification has been achieved, animals and animal wastes are generally concentrated, facilitating disease transmission and water pollution in the absence of proper controls^1^.

On a larger scale, livestock systems have a bi-directional relationship with climate change. Climate change affects the quantity and quality of feeds available to livestock, drives production losses due to heat stress, promotes uneven distribution of water resources, and modifies the distribution of livestock diseases and disease vectors, representing another manifestation of the disproportionate burden climate change places on the world’s resource poor. In turn, livestock and livestock systems are substantial users of natural resources, in particular water and land for grazing and feed production, and contribute to greenhouse gas emissions and climate change^5,8^.

Thus, high spatiotemporal resolution maps of the distribution of livestock hold utility for a range of research and policy applications, from epidemiology and public health to economics and climate science. To this end, the Gridded Livestock of the World (GLW) database was developed in 2007^9^, with GLW-3 being the most recent iteration. Published in 2018 but providing results for 2010, GLW-3 produces two sets of results, one using dasymetric modeling to disaggregate census counts, which is based on weights derived from statistical models that use spatial covariates, and a simple aerial weighting approach that produces estimates free from association with the spatial covariates used in the daysmetric results. These results are produced globally at a resolution of 0.083333$^{\circ }$ for counts of cattle, buffaloes, horses, sheep, goats, pigs, chickens, and ducks^10^. GLW does not use any household survey data, such as the Demographic and Health Survey or the Living Standards Measurement Studies, as these data use complex survey sampling approaches that would not be well-suited to GLW’s current modeling approaches. While numerous prior authors have mapped livestock diseases and disease vectors, beyond GLW mapping of livestock itself remain isolated to highly localized efforts such as grazing intensity in Kazakhstan^11^ and livestock movements in Sahelian Africa^12^.

To build on and support GLW efforts, we have used household survey and census data on livestock ownership and the stochastic partial differential equations (SPDE) approach to Gaussian process modeling to generate high-resolution (0.017$^{\circ }$) maps of cattle and pig density. In contrast to the dasymetric modeling used by GLW, this approach can be applied to both household survey data and census data, expanding the data available for modeling. This approach also leverages spatial structure to strengthen predictions, and generates principled uncertainty estimates derived from posterior distributions. We have produced these maps for every year from 2000–2020 for Malawi and Uganda and 2008–2015 for Democratic Republic of Congo (DRC), facilitating their use in longitudinal analyses. We have additionally used small area estimation to produce density maps at the county level for South Sudan in 2008. In all four countries, we define density as the ratio of animals to humans, producing a map in which the human population is flattened. We present here our methods and resulting products, and discuss our results in the context of GLW-3 and extension of these approaches to other countries and species.

## Results

### Input data

**Figure 1:**
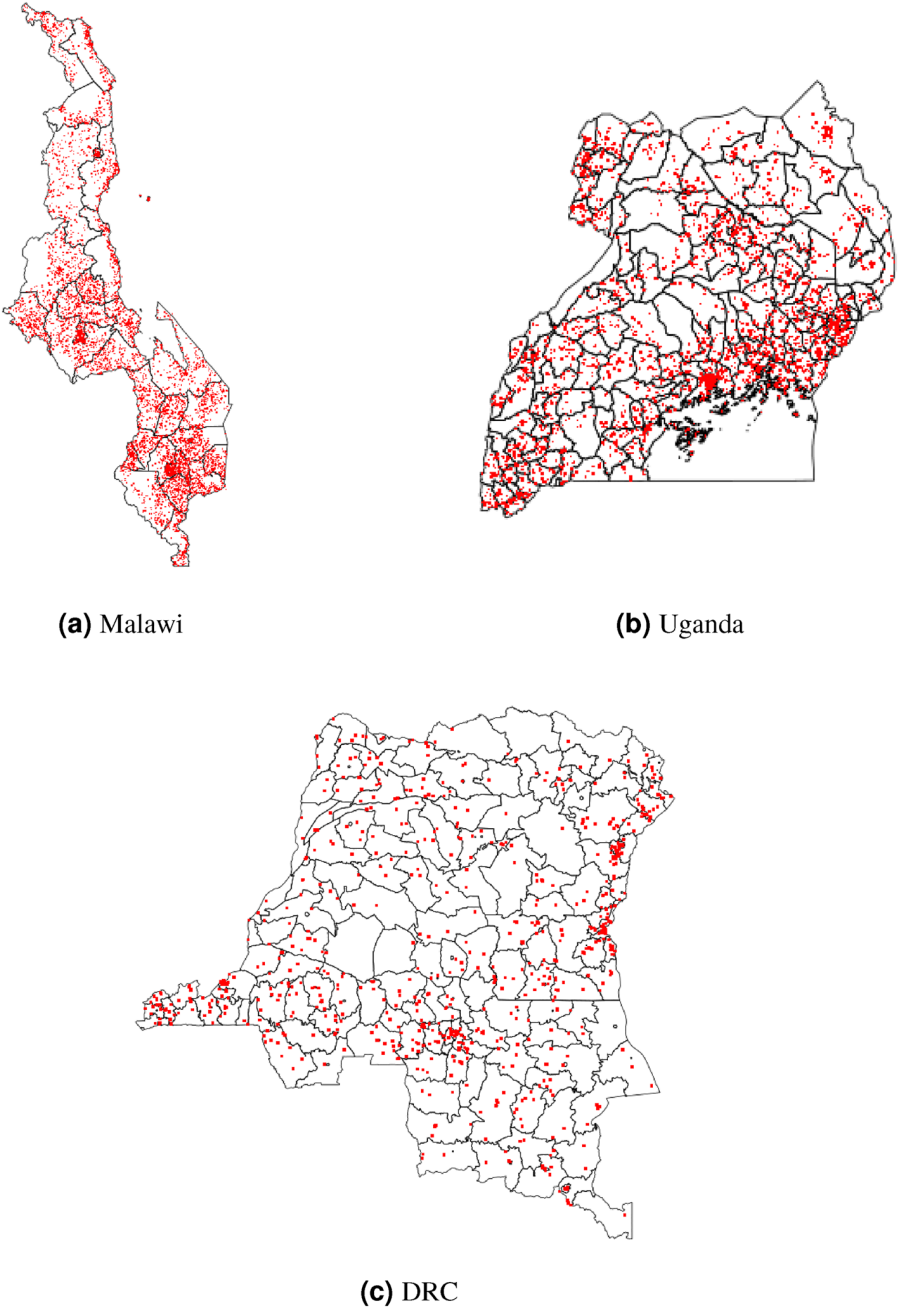
Spatial distribution of survey clusters (observed data) across all years. Size of the red dots does not scale with data richness.

**Figure 2:**
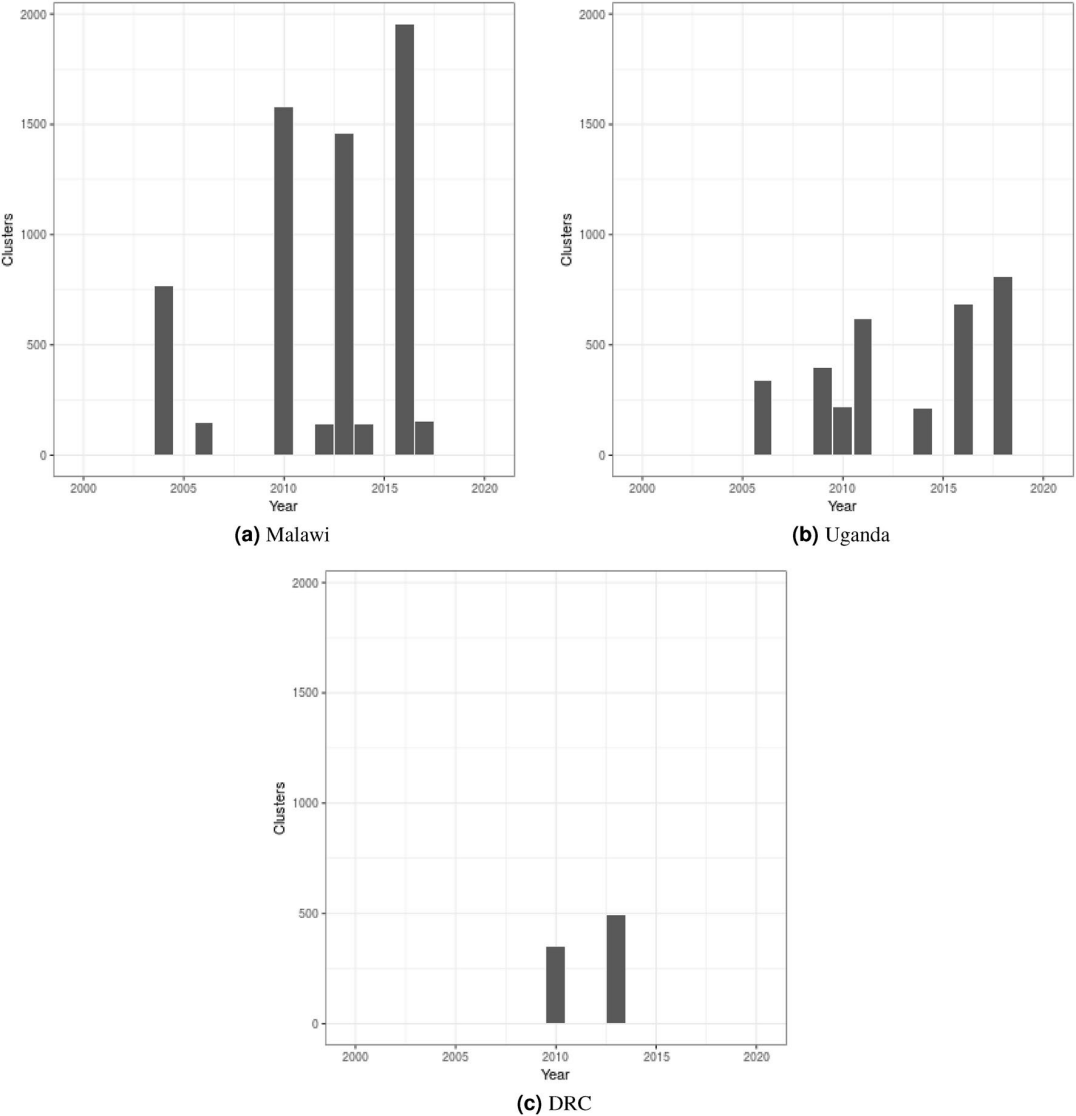
Number of survey clusters (enumeration areas) available for cattle density mapping in each year and country. Results were nearly equivalent for pigs.

In the final dataset there were 6330 clusters for cattle mapping and 6342 clusters for pig mapping in Malawi; 3269 clusters for cattle and 3310 clusters for pigs in Uganda; and 861 clusters for each species in DRC. For South Sudan, data were available for all counties except for Khorflus. For the three SPDE countries (Malawi, Uganda, and DRC), cluster distribution in space is presented in Fig. 1, and data richness (number of survey clusters) by year and country is summarized in Fig. 2.

### Model selection (SPDE only)

**Figure 3:**
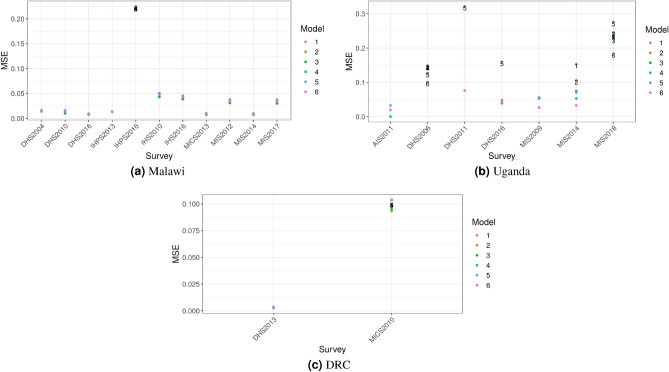
Leave one out cross validation results. Models with mean squared error (MSE) $>0.1$ are labeled. Removed from figures (but not MSE calculations) for interpretablity: (**a**) Malawi: 2004 Integrated Household Survey, all models; Uganda (**b**): 2009 National Panel Survey (NPS), all models, 2011 DHS, models 1-2, 2010 NPS, models 1 and 3-6, and 2011 NPS, models 1-3 and 5.

Leave-one-out cross-validation (LOOCV) results are presented in Fig. 3; we have removed models and surveys with very high mean squared error (MSE) for the sake of interpretability of these figures—as detailed in the accompanying caption—however these surveys and models were included when examining overall model performance. Candidate models differed in fixed effects—covariates—and flexibility of the temporal random effect. For Uganda the best-fitting model was model 6 (MSE 0.076) which included all candidate covariates (urban/rural status, protected areas, elevation, and bodies of water) and a less flexible temporal random effect (random walk 1). For Malawi, model 3 was the best fit (MSE 0.023) which included urban/rural status, protected areas, and a more flexible temporal random effect (random walk 2), and for DRC model 2 (urban/rural status only, random walk 2) was the best fit (MSE 0.048).

In all three countries, we found extremely strong positive associations between our livestock density estimates and those derived from GLW-3 (coefficients greater than 100), for both cattle and pig density, after using WorldPop^13^ data as a denominator to convert livestock counts estimated by GLW-3 to densities. These remarkably high coefficients can be explained by the coarser resolution of GLW-3 (0.083$^{\circ }$) compared with both our maps (0.017$^{\circ }$) and WorldPop data (0.0083$^{\circ }$), resulting in markedly higher density estimates for GLW-3. They do, however, indicate general agreement between our estimates and those of GLW-3.

### Random effects posteriors

For the marginal standard deviation, the selected prior yields a posterior 95% credible interval of (0.37, 2.72) for the residual rate ratio (deviations in livestock density above or below the mean model). For the iid random effects, the specified priors result in a posterior 99% credible interval for the residual variance of (0.6, 1.6).

### Maps

**Figure 4:**
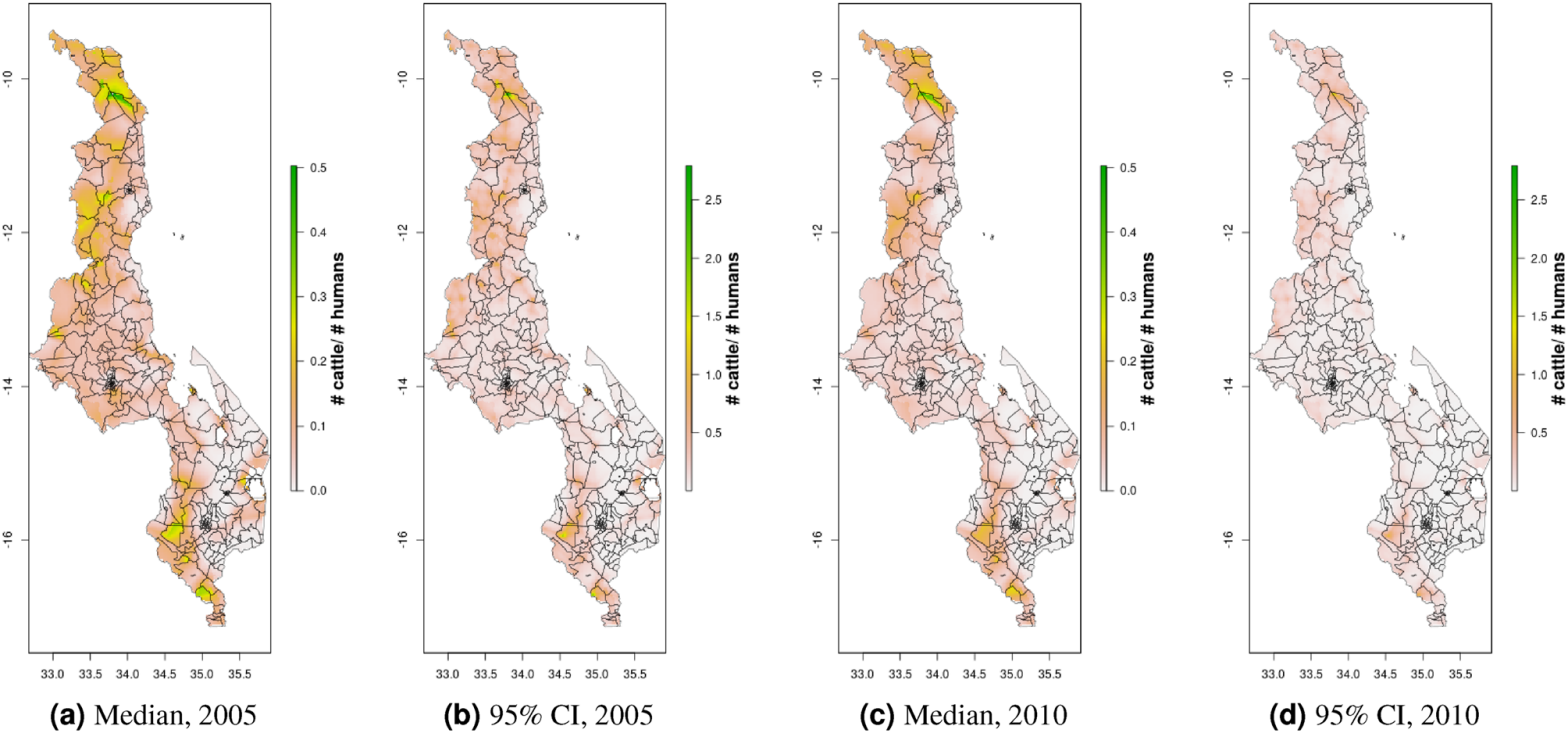
Cattle density mapping results, Malawi, 2005 and 2010. Median (**a, c**) and width of posterior 95% credible interval (**b, d**).

**Figure 5:**
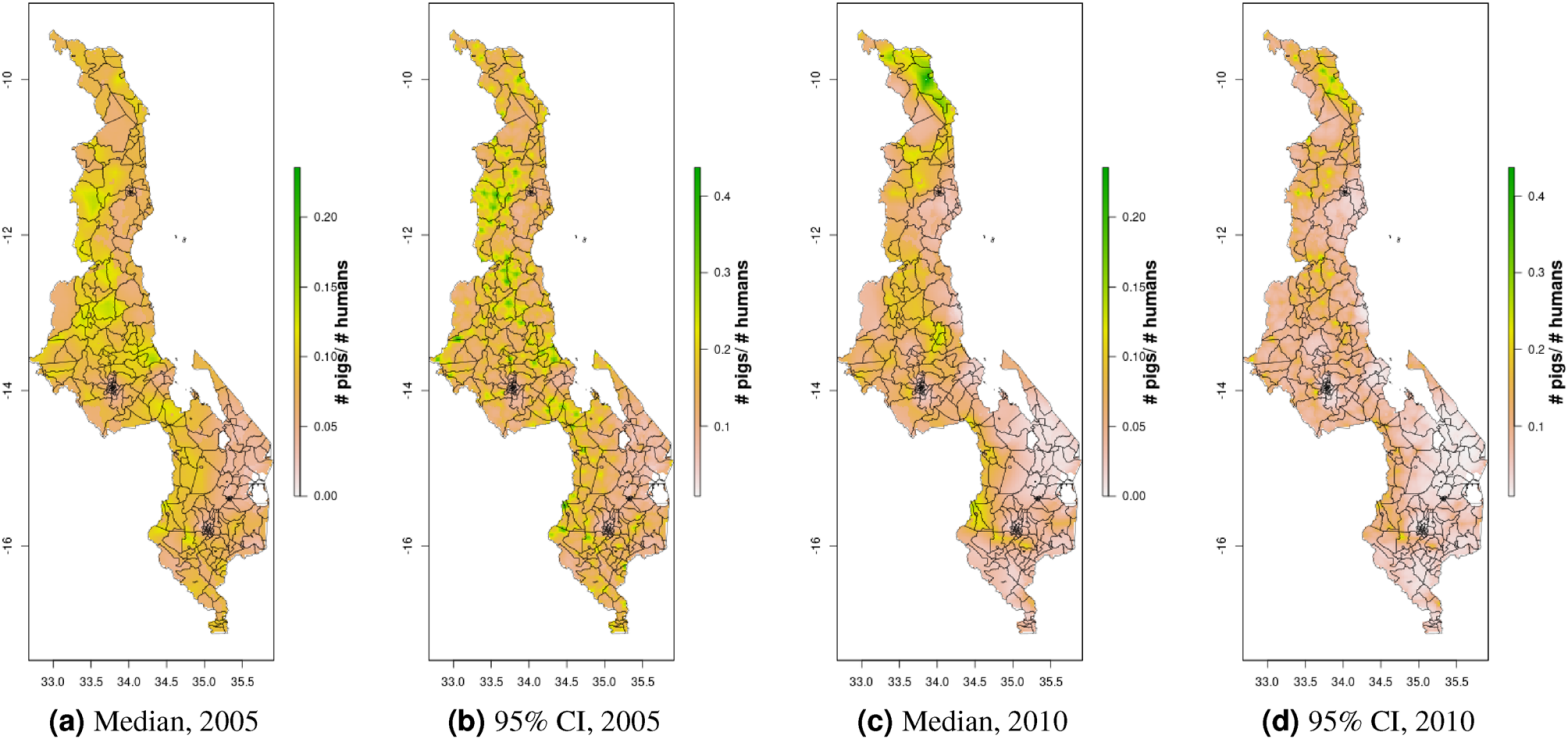
Pig density mapping results, Malawi, 2005 and 2010. Median (**a, c**) and width of posterior 95% credible interval (**b, d**).

**Figure 6:**
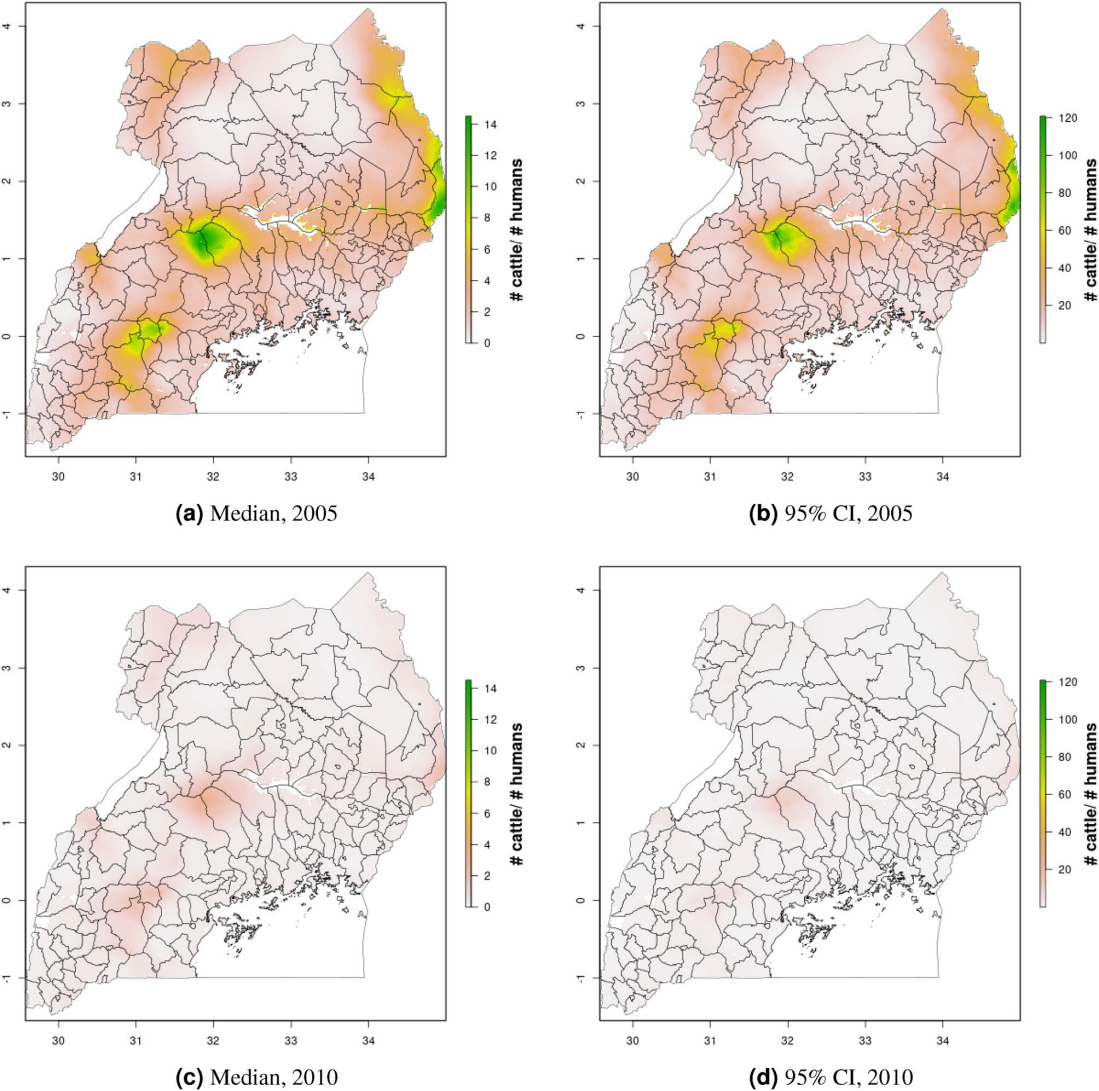
Cattle density mapping results, Uganda, 2005 and 2010. Median (**a, c**) and width of posterior 95% credible interval (**b, d**).

**Figure 7:**
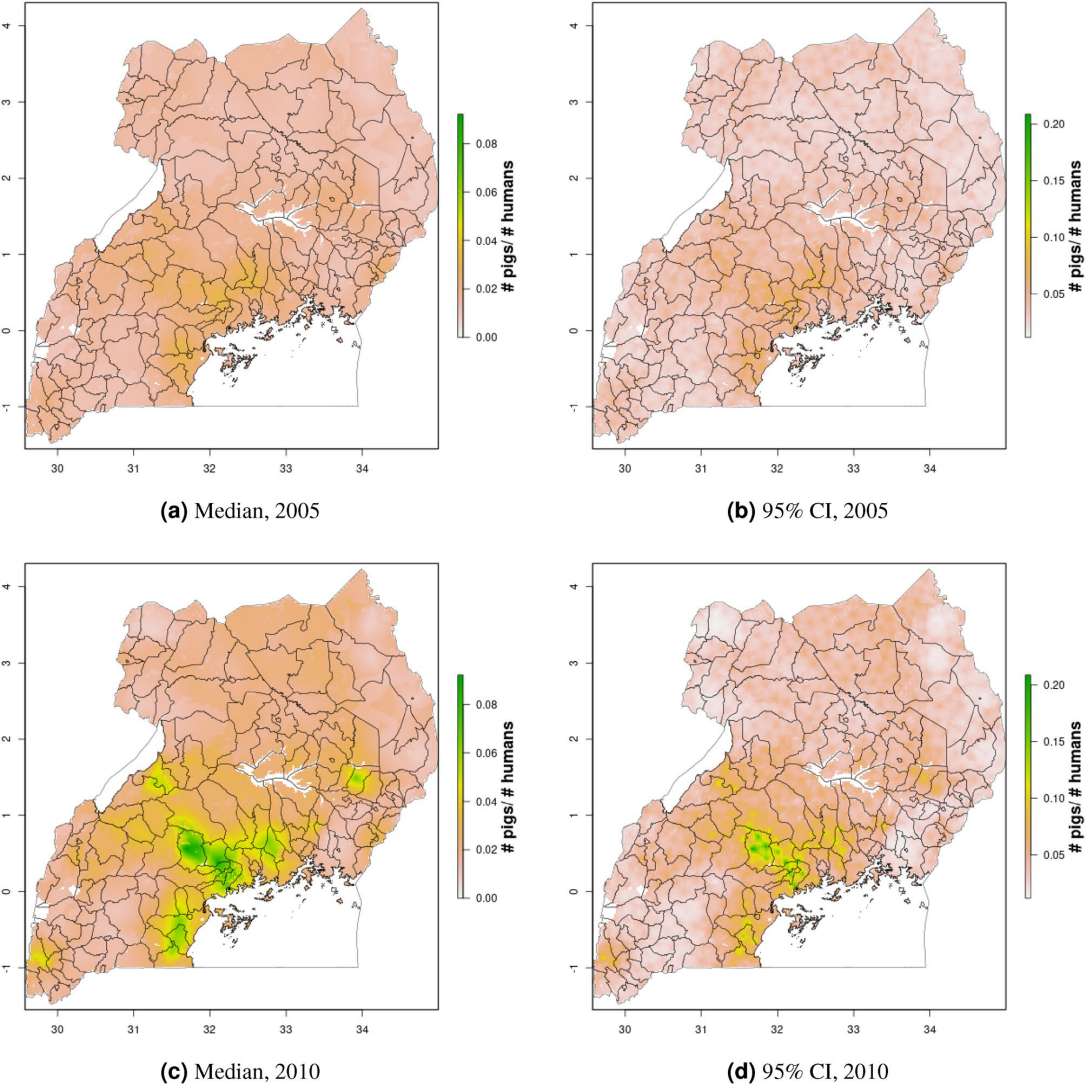
Pig density mapping results, Uganda, 2005 and 2010. Median (**a, c**) and width of posterior 95% credible interval (**b, d**).

**Figure 8:**
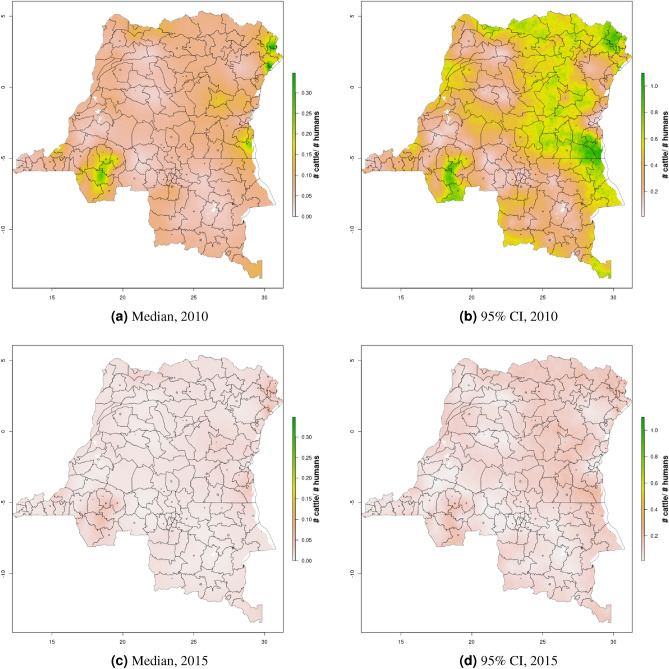
Cattle density mapping results, DRC, 2010 and 2015. Median (**a, c**) and width of posterior 95% credible interval (**b,d**).

**Figure 9:**
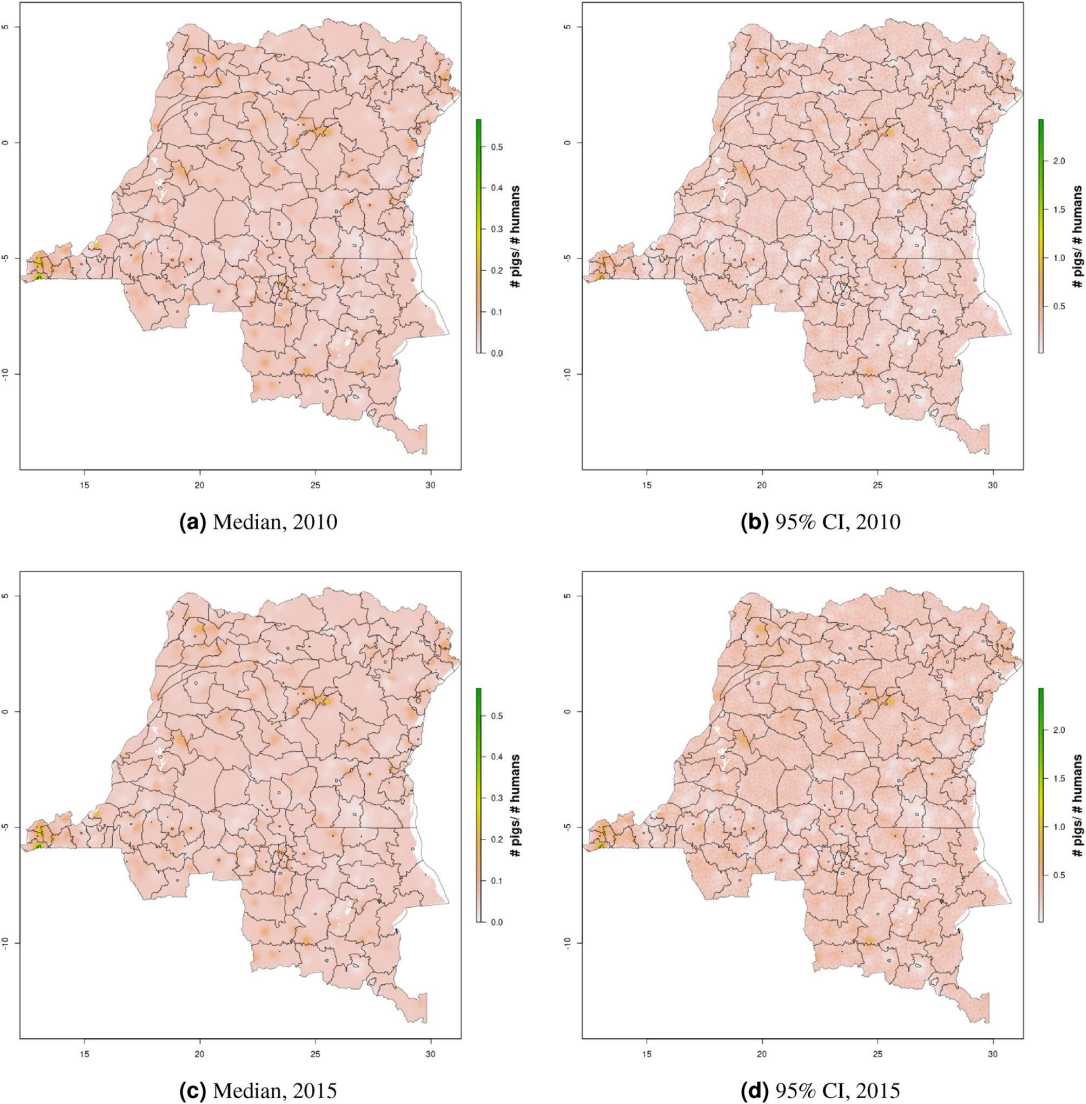
Pig density mapping results, DRC, 2010 and 2015. Median (**a, c**) and width of posterior 95% credible interval (**b, d**).

**Figure 10:**
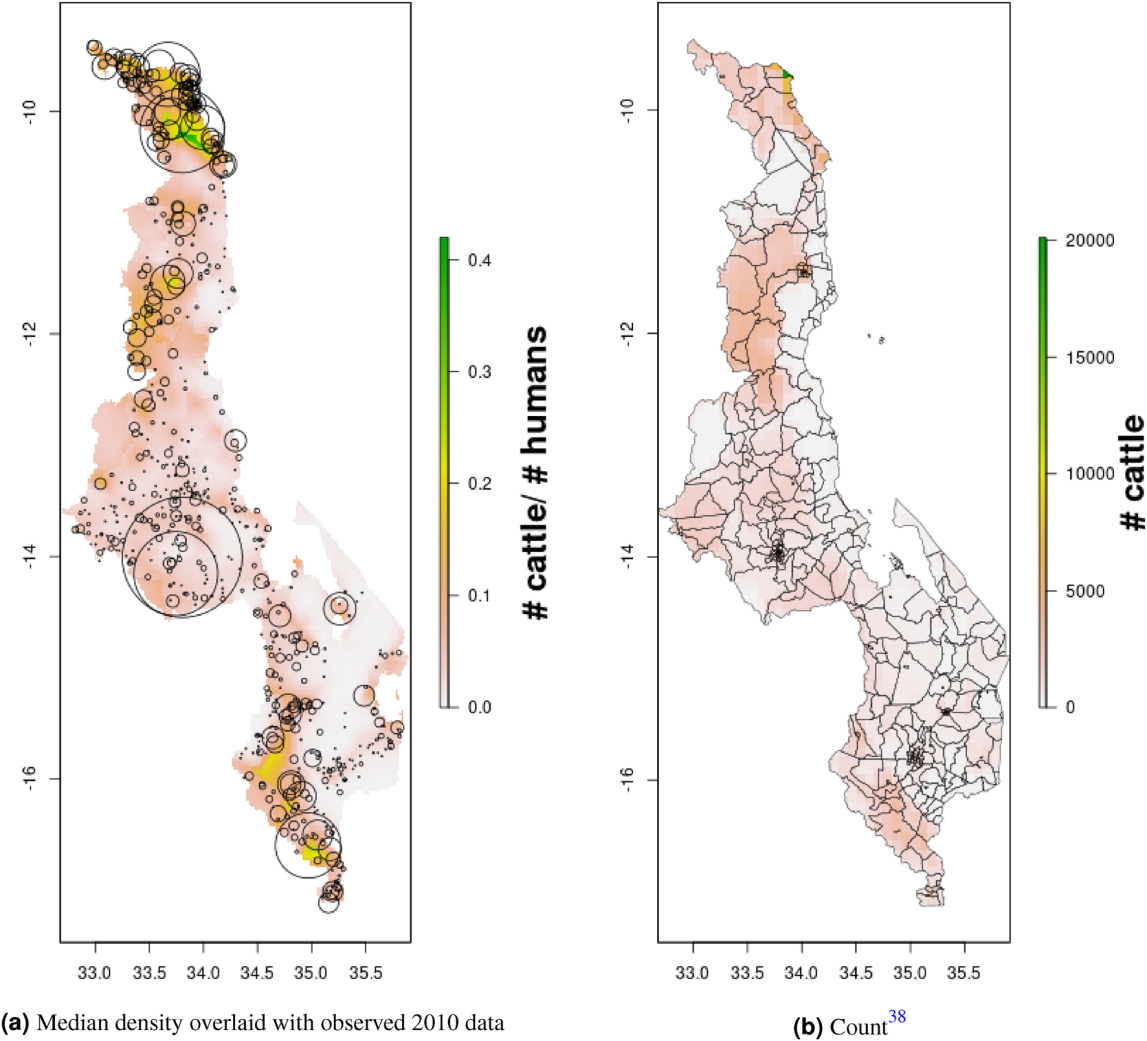
Median cattle density (L) compared with estimated cattle counts produced by GLW-3 (R) for Malawi, both 2010.Observed density data for 2010 are presented on the left, indicated by circle size; this figure does not represent the contribution of data from other years to the model for 2010 via the temporal random effect, nor the contribution from predictors^16^.

**Figure 11:**
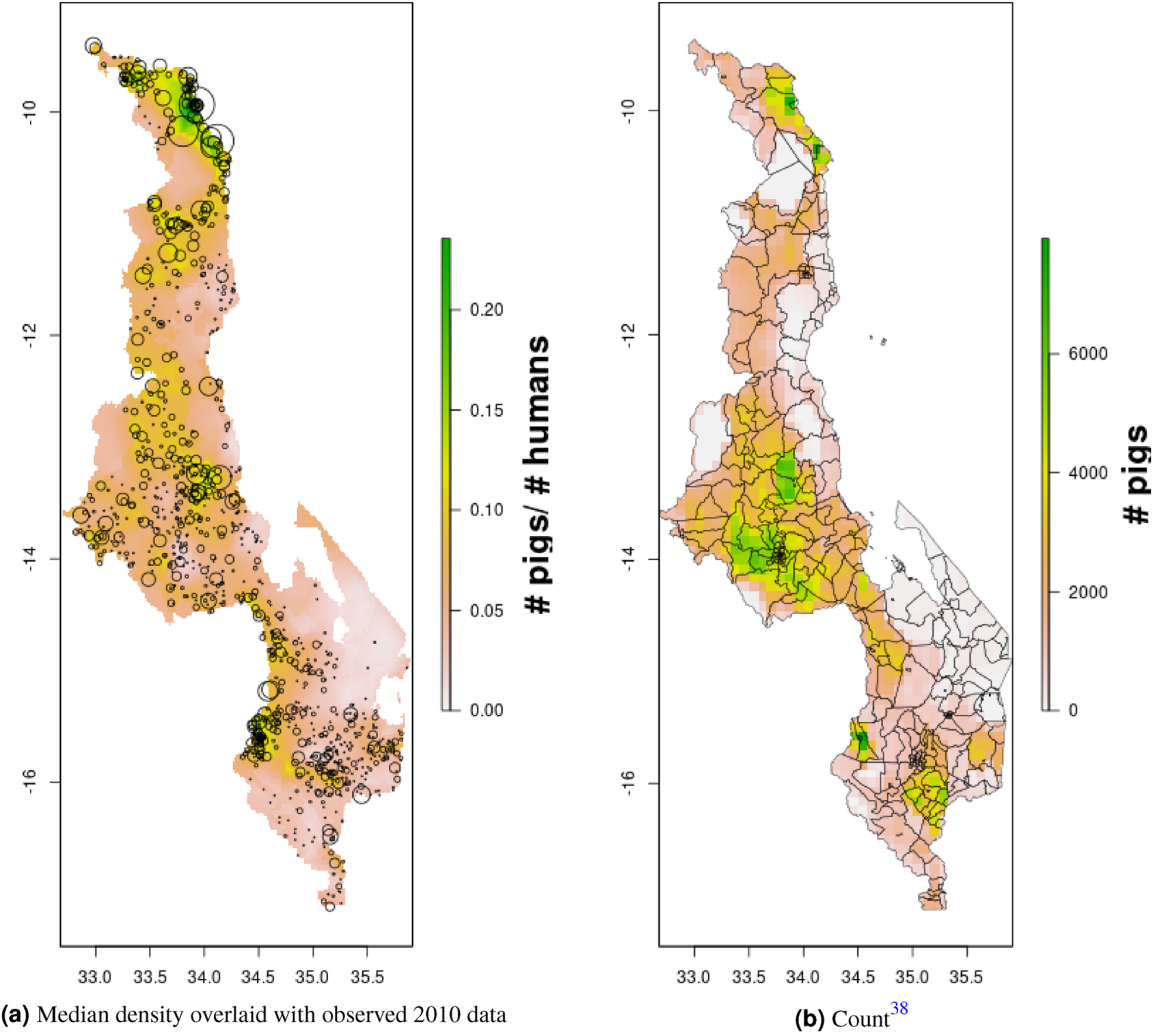
Median pig density (L) compared with estimated pig counts produced by GLW-3 (R) for Malawi, both 2010.Observed density data for 2010 are presented on the left, indicated by circle size; this figure does not represent the contribution of data from other years to the model for 2010 via the temporal random effect, nor the contribution from predictors^16^.

**Figure 12:**
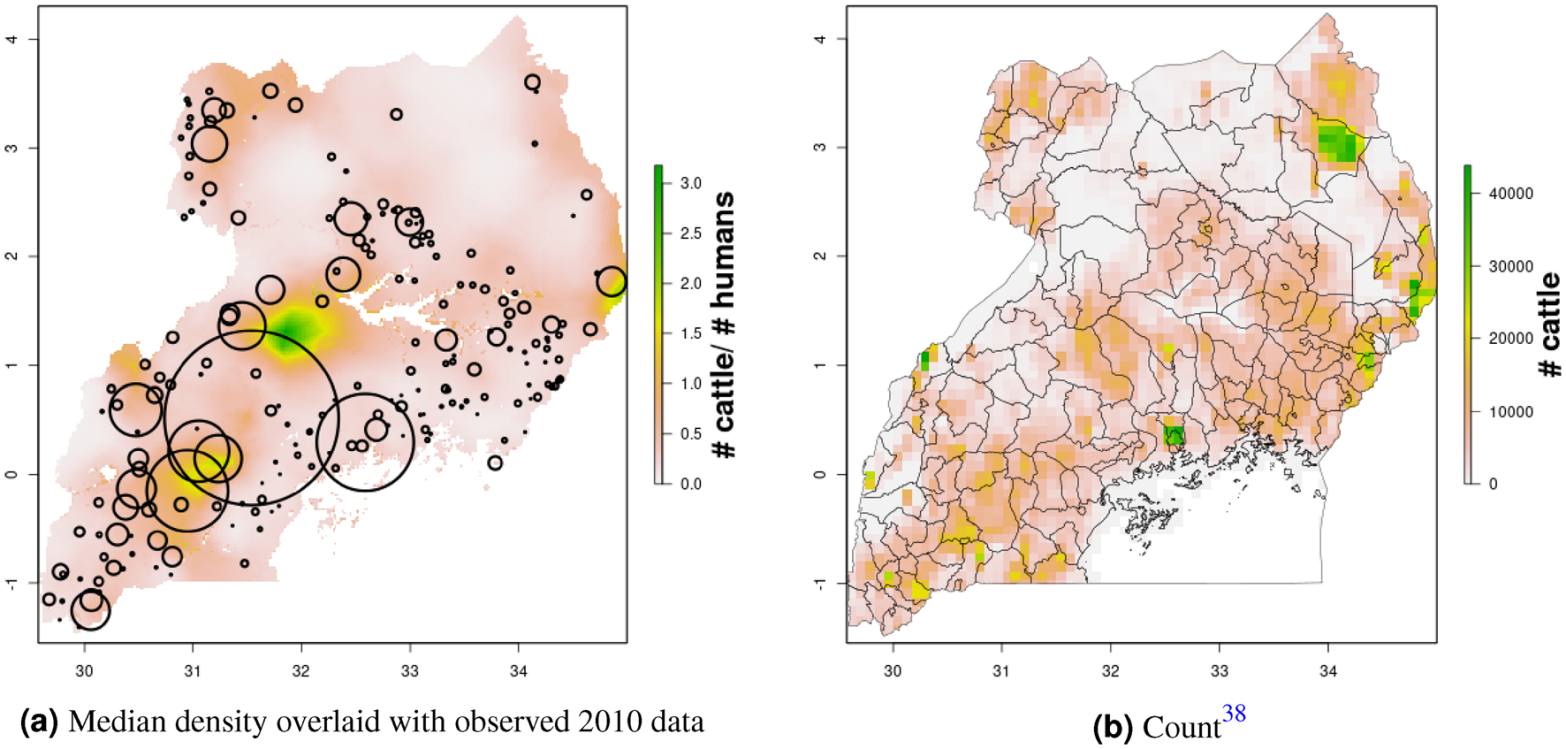
Median pig density (L) compared with estimated pig counts produced by GLW-3 (R) for Uganda, both 2010. Observed density data for 2010 are presented on the left, indicated by circle size; this figure does not represent the contribution of data from other years to the model for 2010 via the temporal random effect, nor the contribution from predictors^16^.

**Figure 13:**
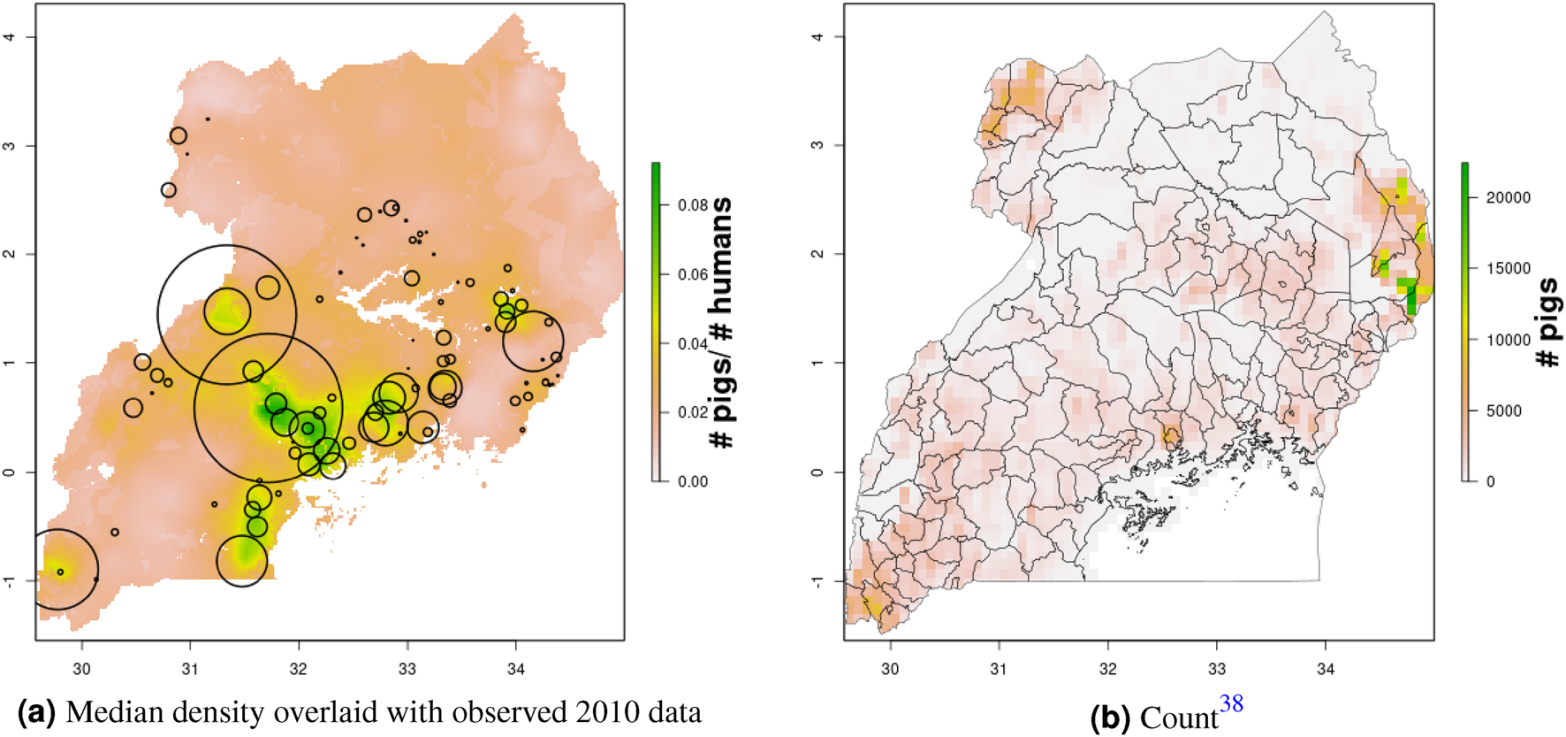
Median cattle density (L) compared with estimated cattle counts produced by GLW-3 (R) for DRC, both 2010. Observed density data for 2010 are presented on the left, indicated by circle size; this figure does not represent the contribution of data from other years to the model for 2010 via the temporal random effect, nor the contribution from predictors^16^.

**Figure 14:**
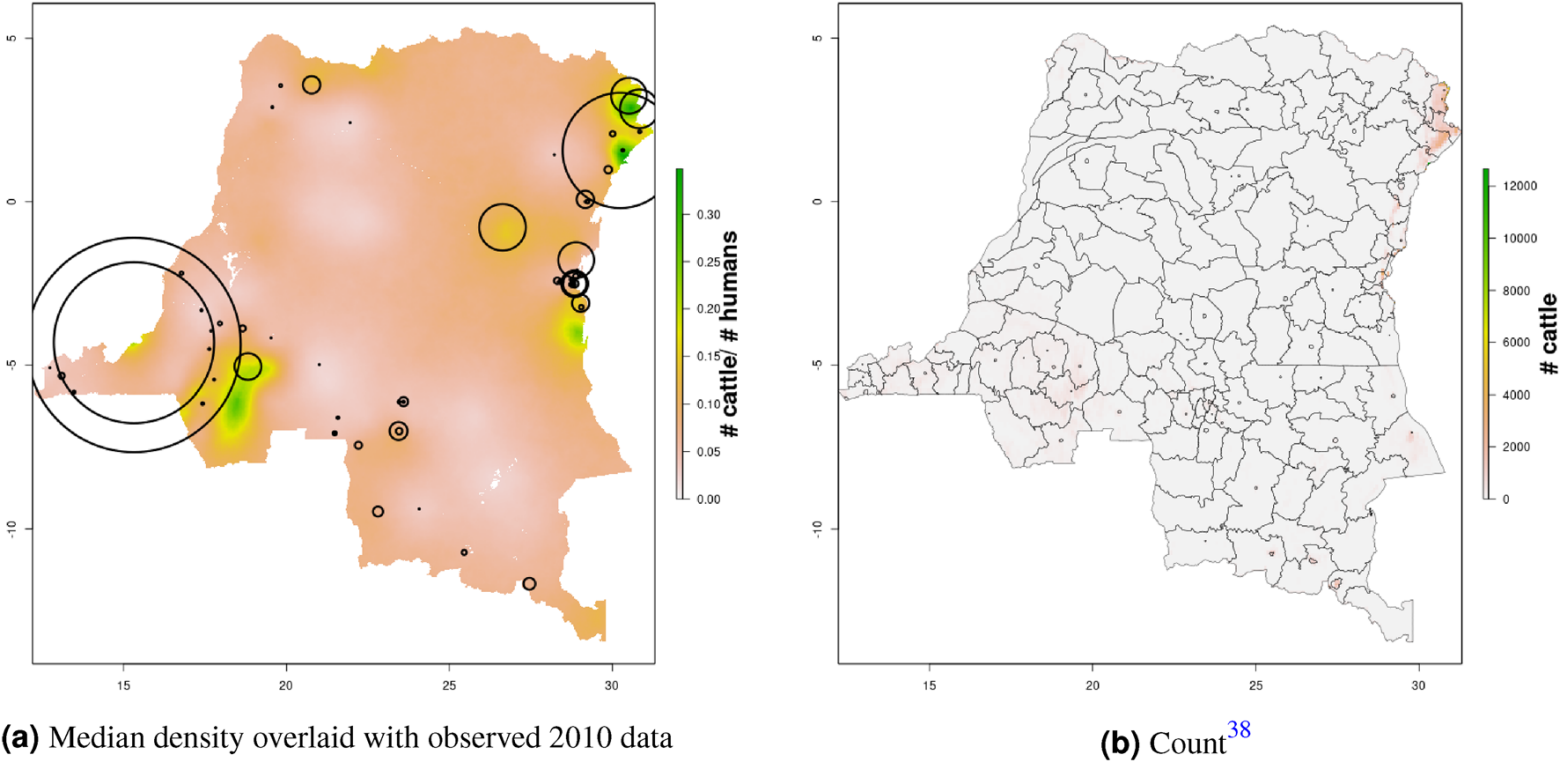
Median cattle density (L) compared with estimated cattle counts produced by GLW-3 (R) for DRC, both 2010.Observed density data for 2010 are presented on the left, indicated by circle size; this figure does not represent the contribution of data from other years to the model for 2010 via the temporal random effect, nor the contribution from predictors^16^.

**Figure 15:**
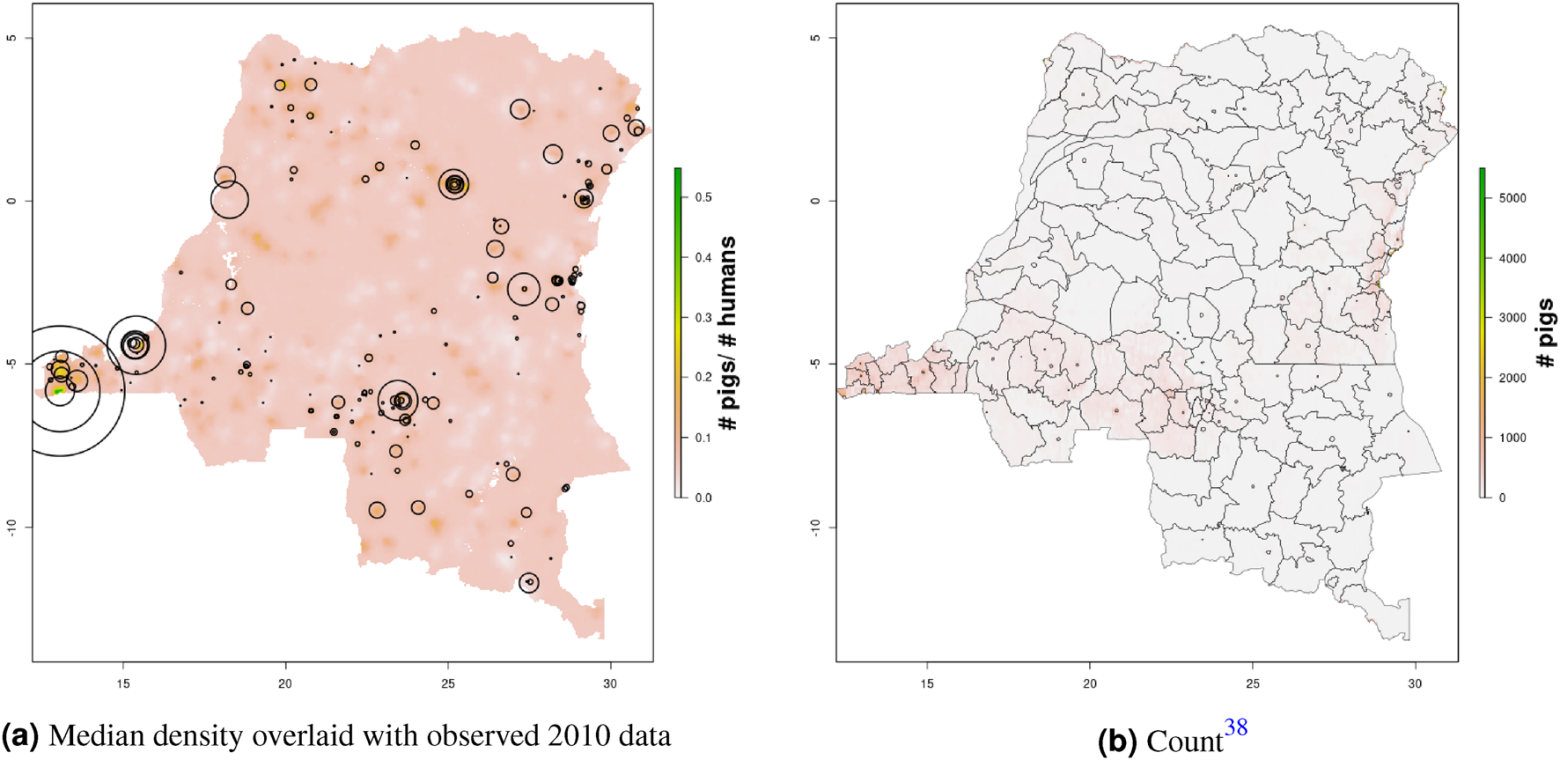
Median pig density (L) compared with estimated pig counts produced by GLW-3 (R) for DRC, both 2010.Observed density data for 2010 are presented on the left, indicated by circle size; this figure does not represent the contribution of data from other years to the model for 2010 via the temporal random effect, nor the contribution from predictors^16^.

**Figure 16:**
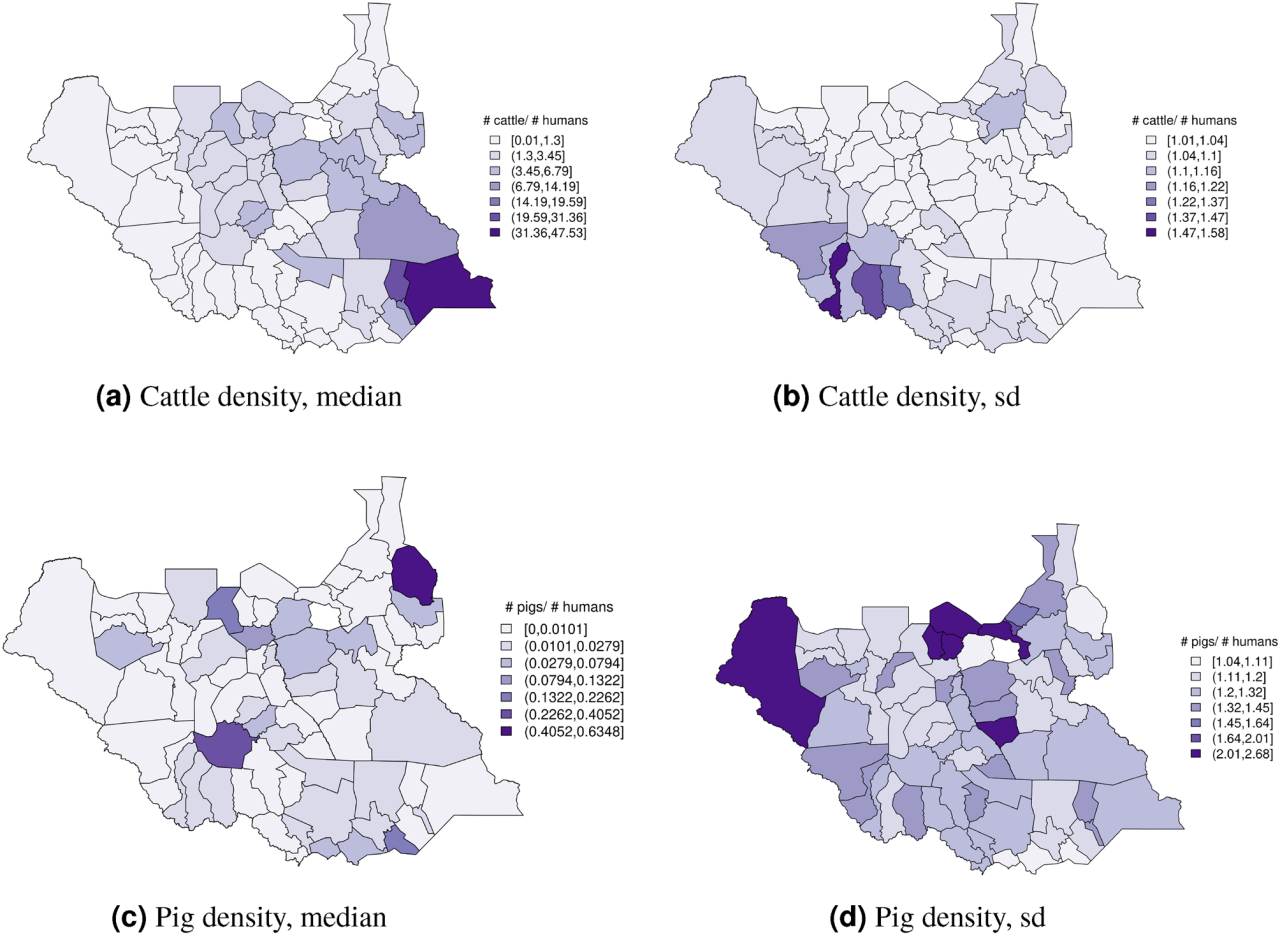
Cattle (**a-b**) and pig (**c-d**) density mapping results, South Sudan, 2008. Median (**a, c**) and standard error (**b, d**).

**Figure 17:**
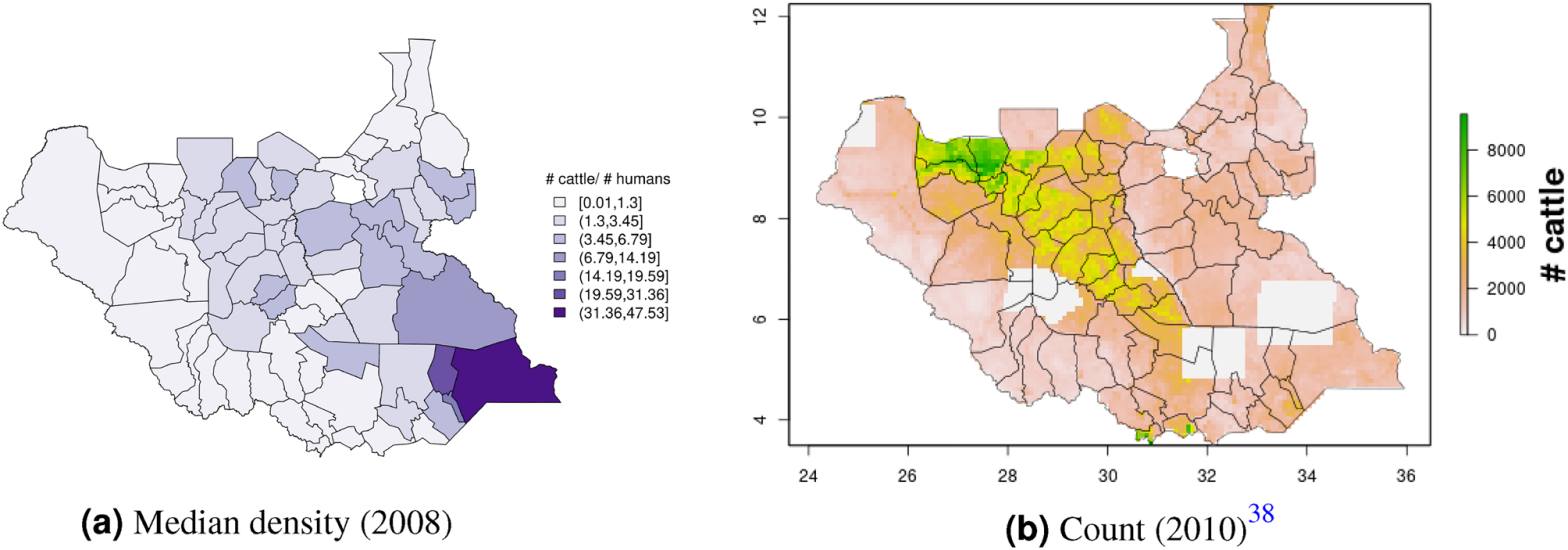
Median cattle density (L, 2008) compared with estimated cattle counts produced by GLW-3 (R, 2010) for South Sudan. Observed density data for 2010 are presented on the left, indicated by circle size; this figure does not represent the contribution of data from other years to the model for 2010 via the temporal random effect, nor the contribution from predictors^16^.

**Figure 18:**
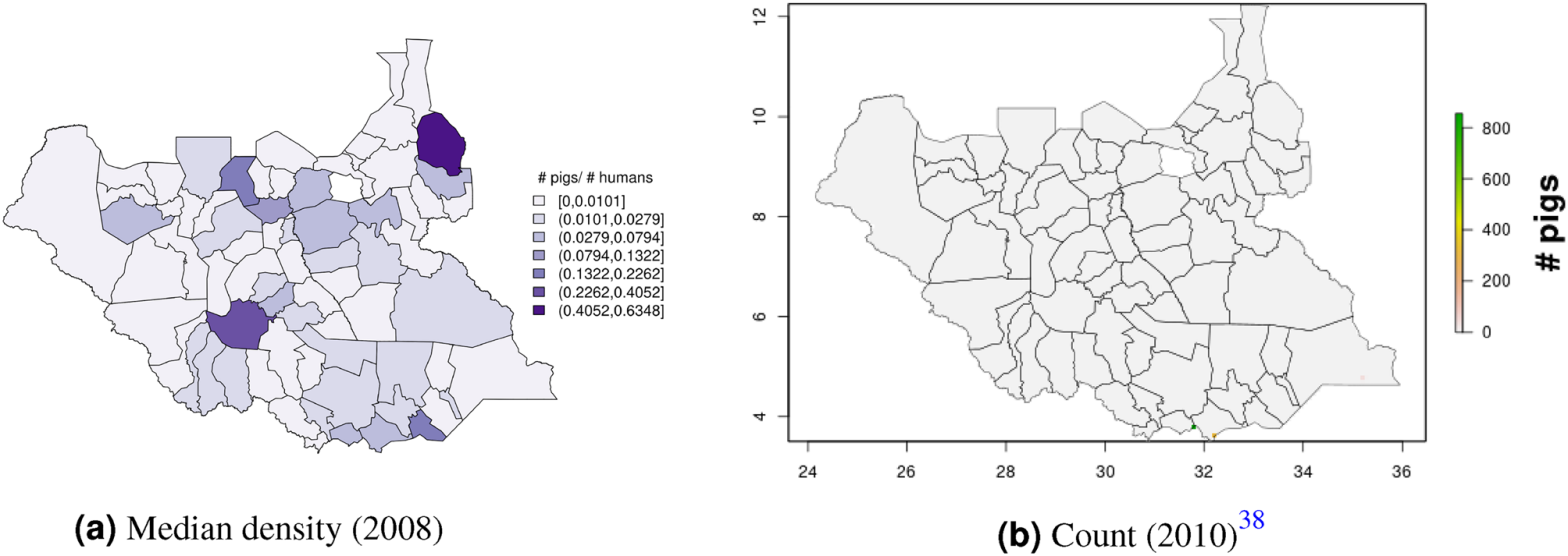
Median pig density (L, 2008) compared with estimated pig counts produced by GLW-3 (R, 2010) for South Sudan^16^.

Density maps for 2005 and 2010 for Malawi and Uganda, 2010 and 2015 for DRC, and for 2008 for South Sudan, are presented in Figs. 4, 5, 6, 7, 8, 9, 16. Maps for 2010 are presented next to corresponding GLW-3 products in Figs. 10, 11, 12, 13, 14, 15, and 17, 18. Results (median and width of the posterior 95% credible interval) as .tif files for every year for Malawi, Uganda, and DRC, and as shapefiles for 2008 for South Sudan, are available in a GitHub repository: https://github.com/JulianneMeisnerUW/LivestockMaps. Providing estimates of both median and uncertainty allows users to assess the quality of the estimates for each species, country, and year. All R code used to generate these maps are also in this repository.

In Malawi (Figs. 4, 5), cattle density was highest in the northern and southern extents of the country, and pig density generally decreased along a west-east gradient. Across all years and clusters, median cattle density was 0.05, and median pig density was 0.07. Per FAOSTAT total stock estimates^14^ and World Bank national population estimates^15^, mean national cattle density over the period 2000-2019 was 0.08, and mean national pig density was 0.15. Spatial patterns appear to largely agree across our maps and GLW-3 maps cropped to Malawi (Figs. 10, 11); exact agreement is not expected as these maps are parameterized differently.

In Uganda (Figs. 6, 7), the “cattle corridor” is clearly visible in both years, while pig density appears to be higher in the south of Uganda. Across all years and clusters, median cattle density was 0.23, and median pig density was 0.02. Per FAOSTAT total stock estimates^14^ and World Bank national population estimates^15^, mean national cattle density over the period 2000–2019 was 0.33, and mean national pig density was 0.07. Spatial patterns are similar to those of GLW-3 cropped to Uganda (Figs. 12, 13), however two GLW-3 hotspots—a cattle density hotspot in the northeast and a pig density hotspot in the eastern part of the northern region—were not found in our maps, and pig density hotspots in the Central Region detected in our maps was not detected in GLW-3; again, we do not expect perfect agreement between these products.

In DRC, there are “hotspots” of cattle density in the east and southwest of the country, and no obvious spatial patterns for pig density (Figs. 8, 9) Per FAOSTAT total stock estimates^14^ and World Bank national population estimates^15^, mean national cattle density over the period 2008–2015 was 0.013 for cattle and 0.014 for pigs, while across all years and clusters our median estimate was 0.037 for cattle and 0.053 for pigs. Spatial patterns are difficult to detect in the GLW-3 cattle product for DRC (Fig. 14), however a density hotspot in the northeast is evident in both maps. In the pig maps, a density hotspot in west-central DRC that is evident in both products (Fig. 15).

Notably, in Malawi, DRC, and Uganda, a general decrease in cattle density is observed over time across the two plotted years (2005 and 2010 for Uganda and Malawi, 2010 and 2015 for DRC). For pigs this is observed to a much lesser extent in Malawi, and not at all in Uganda and DRC. This likely represents an increase in human population density that is matched by corresponding increases in pig density but not cattle density. Figures 10, 11, 12, 13, 14, 15 also present modeled and observed density for 2010 in each country. Agreement appears good overall, with the apparent exception of cattle in Uganda (Fig. 12), where several observed foci of high density are not captured in the modeled surface. This can likely be attributed to observations from adjacent years (2009 and 2011), which will influence 2010 estimates through the temporal random effect, and are not represented in this time stratified figure.

In South Sudan (Fig. 16), cattle density was on average higher than pig density, and there is a clear decreasing southeast-northwest trend in cattle density. For pig density in South Sudan, other than generally low densities in the northwest of the country, there are no clear spatial trends. Across all counties, median density was 1.56 for cattle and 0.007 for pigs in 2008. Per FAOSTAT total stock estimates^14^ and World Bank national population estimates^15^, mean national cattle density over the period 2000–2019 was 1.33; no FAOSTAT entry is available for pigs in South Sudan. On comparison with GLW-3 maps cropped to South Sudan, “hotspots” of cattle density are more to the east in our maps than GLW-3 maps (Fig. 17), and pig density is markedly different in our maps from count distribution in GLW-3 (Fig. 18).

**Figure 19:**
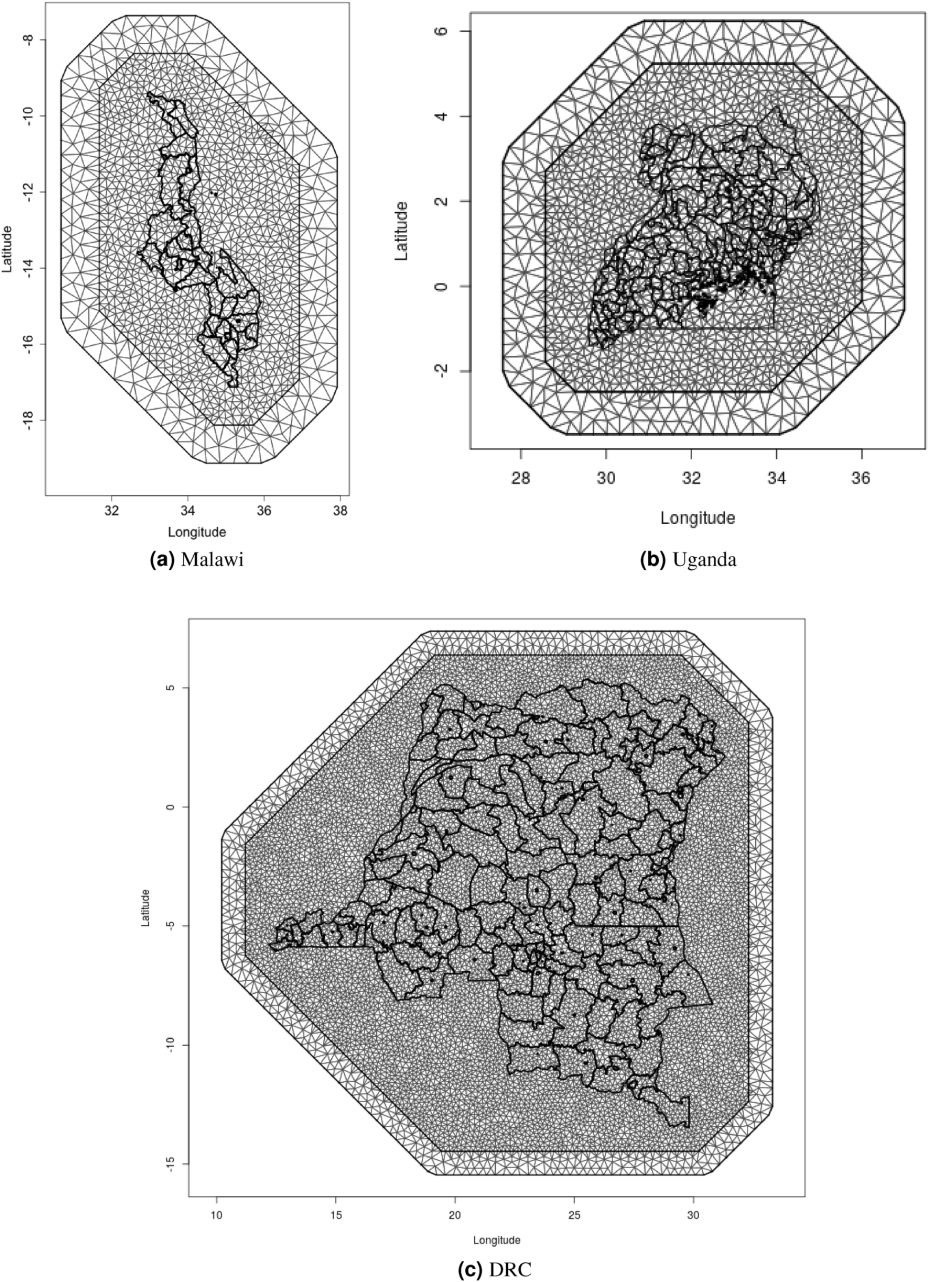
Meshes used for SPDE.

## Discussion

We have generated a set of cattle and pig density maps for Malawi, DRC, Uganda, and South Sudan, publicly available for use in economic, public health, environmental, agricultural, and other applications. Our results compliment GLW-3 efforts by producing a longitudinal product in Malawi, DRC, and Uganda, parameterizing livestock as the ratio of heads of cattle and pigs to the human population, and utilizing distinct input data and an entirely different modeling approach.

**Figure 20:**
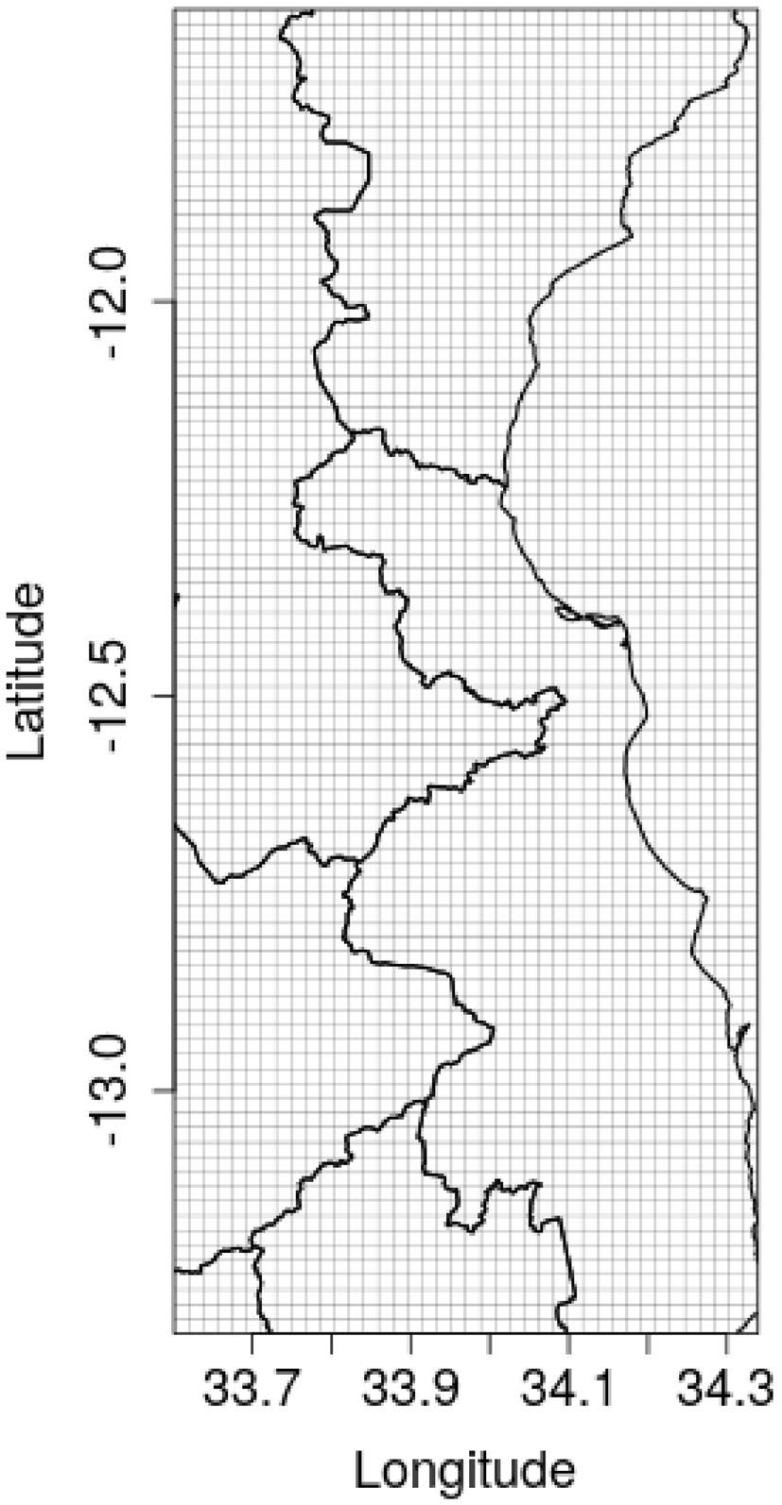
Regular grid for prediction, cropped to the northwest of Malawi. Prediction grids were of equivalent resolution in Uganda and DRC.

In contrast with GLW-3, we did not mask any pixels as unsuitable for livestock. The result is our findings impose no assumptions with regards to livestock presence in urban and high-altitude pixels, livestock incursion into protected areas, and wet versus dry season changes in the distribution of water bodies, but may result in bias in pixels corresponding to large and permanent bodies of water (Fig. 20).

There are several limitations to our results. First, there may be inaccuracies in the input data, for instance over-reporting of livestock ownership due to the social desirability of owning a larger herd, or under-reporting due to concerns about increased taxation. Other inaccuracies may arise due to jittering in the input data, in which clusters in publicly-available datasets are randomly displaced to protect participant privacy. In light of the high resolution of our product (approx. 2 km$^2$ at the equator), this is particularly problematic in rural areas, where displacement is up to 5 km in DHS data, and a random 1% of clusters are displaced up to 10 km^17^. To our knowledge methods for probabilistically “un-jittering” data are under development but not yet available. While ground-truthing is both cost-prohibitive and impractical for a longitudinal product, our LOOCV results indicate low MSEs overall, and good agreement between our results and GLW-3 results is a promising indicator of the validity of both products. Furthermore, our findings generally agreed with national density estimates, and we would not expect perfect agreement with such estimates as local distribution patterns for humans is unlikely to be equivalent to local distribution patterns for livestock, and we are comparing medians to means. In addition to jittering in the input data sources, misclassification of livestock density may arise from the location definition used in our models, whereby livestock are assigned to the centroid of the corresponding enumeration area. Thus our maps should be interpreted as the density of livestock to humans when both are in their place of residence (i.e., at nighttime). While this may be a poor reflection of livestock presence in pastoralist systems, it is difficult to conceptualize any other definition of livestock density as the daily movements of humans and livestock may be distinct.

Second, our results treat livestock production systems as equivalent within a given species, however the environmental, socioeconomic, and public health relevance of production system types (e.g., pastoralist vs. intensive) are distinct, potentially reducing the utility of our results for select uses. Furthermore, users will need to take care to ensure the predictors (spatial covariates) used in our SPDE models do not introduce circularity in their analyses. We have opted for a parsimonious (few covariates) model and excluded vegetation indices as a predictor for this reason, however, users who wish to study the distribution of livestock with respect to, for instance, national parks, may find use of our maps biases their results, as boundaries of protected areas were used as a predictor in our model.

Third, our approach to generating urbanicity surfaces reflects the challenging nature of meaningfully assigning unsampled cluster-years as urban versus rural. Our approach performed well in the test data with the exception of urban clusters in Uganda, for which it performed very poorly. Alternative approaches, such as using Global Human Settlement Layer data^18^ and other existing urbanicity surfaces, do not necessarily correspond to the definition of urbanicity needed in our application, nor are they generally available as a longitudinal product.

Fourth, due to limited data in DRC and South Sudan, we could not include a space-time interaction term in DRC—imposing the assumption that spatial trends are constant in time, and temporal trends are constant in space—and could only produce a county-level map for South Sudan, and only for a single year.

Finally, our estimates are accompanied by uncertainty which varies across time, countries, and clusters. Uncertainty was highest for DRC and for early years, rendering our maps less useful in these cluster-years. It is our hope that by producing uncertainty estimates alongside of medians, it will be transparent to users where our results are more or less stable.

For poor livestock keepers throughout the world, livestock represent a lifeline, providing financial security, nutrition, transportation, and connection to cultural identities. Livestock, however, also represent a source of exposure to zoonoses, and a driver of environmental change on local and global scales. To grapple with these divergent effects in research and policy, high-resolution estimates of livestock distribution tethered to human population distribution are needed. We present such a product, which, despite its limitations, represents an important extension to GLW-3 with high demonstrated validity and broad potential utility.

## Methods

### Data collection and processing

Data sources were identified by searching GHDx and the IHSN Central Data Catalog^19,20^, and microdata were downloaded from publicly-accessible websites or by request to relevant national agencies. Both Demographic and Health Survey (DHS) data and population and housing census data were downloaded from IPUMS^21,22^; most other surveys were downloaded from links in the relevant IHSN entry. Data were processed by source; data sources by country are detailed in Table 1.

**Table 1:** Data sources by country.

Country	Year	Source
Malawi	2004	Second integrated household survey
	2004–2005	Demographic and health survey
	2006	Technology adoption and risk initiative survey
	2010	Demographic and health survey
	2010–2011	Third integrated household survey
	2010, 2013, 2016	Integrated household panel survey
	2012	Malaria indicator survey
	2013–2014	Multiple indicator cluster survey
	2014	Malaria Indicator Survey
	2015–2016	Demographic and health survey
	2016–2017	Fourth integrated household survey
	2017	Malaria indicator survey
South Sudan	2008	Population and housing census
DRC	2010	Multiple indicator cluster survey
	2013–2014	Demographic and health survey
Uganda	2006	Demographic and health survey
	2009	Malaria indicator survey
	2009–2010	National panel survey
	2010–2011	National panel survey
	2011	AIDS indicator survey
	2011	Demographic and health survey
	2011–2012	National panel survey
	2014–2015	Malaria indicator survey
	2016	Demographic and health survey
	2018	Malaria indicator survey

As data were only available for a single year for South Sudan (2008), no longitudinal mapping could be performed for this country. Furthermore, these data provided geolocation only to the level of the county (administrative level 2), thus we have used small area estimation, rather than the SPDE approach, to generate county-level maps in South Sudan. In Malawi, Uganda, and DRC, survey cluster—the smallest geographical sampling unit of a household survey, typically an enumeration area consisting of approximately 100 households—was the unit of analysis.

Number of livestock was parameterized as number in sampled households, summed over cluster (Malawi, Uganda, DRC) or county (South Sudan). For cattle, counts were summed over local and exotic breeds, and dairy and non-dairy herds. Similarly, number of humans was parameterized as number of individuals (adults and children) in sampled households, summed over households in a given cluster (Malawi, Uganda, DRC) or county (South Sudan). In Malawi, DRC, and Uganda, clusters with missing values for cattle counts did not contribute to the denominator for cattle density, clusters with missing values for pig counts did not contribute to the denominator for pig density, and clusters missing both pig and cattle counts were removed. As surveys which used stratified sampling methods defined strata by geography and urban/rural status, urban/rural status was also extracted for later modeling. Year was defined as survey year; for surveys conducted over two years, the first year was used. These attribute data were then merged with location data. Finally, source-specific data were merged to create one spatial points dataset (Malawi, Uganda, DRC) or spatial polygon dataset (South Sudan), and predictor data were extracted (detailed below).

### Model fitting

#### Malawi, Uganda, and DRC

For Malawi, Uganda, and DRC, we used the SPDE approach to Gaussian process (GP) modeling. The goal of this approach is to smooth over spatial point data and generate a complete surface.

SPDE approach A spatial process $S({{\textbf {s}}})$ is a GP if the joint distribution of $S({{\textbf {s}}}_1),\ldots ,S({{\textbf {s}}}_n)$ (over the whole study area) is an *n* dimensional Gaussian distribution for any integer *n* and any set of locations ${{\textbf {s}}}_i$ (e.g., defined by latitude and longitude). That is, the spatial process at each location has a Gaussian distribution.

In Bayesian hierarchical spatial modeling, spatial dependence is generally represented by a precision matrix (inverse of a variance-covariance matrix), with the Matérn family being a popular choice of covariance function^23^. It can be shown that Matérn GPs can be represented as the solution an SPDE, but for a GP with *n* clusters, a *n* dimensional normal distribution must be modeled, which quickly becomes computationally intractable. These continuous GPs can be discretized (approximated) via a finite element approach using a weighted combination of basis functions. If the weights are multivariate Gaussian and picked to have a sparse precision matrix (many 0 entries), the resulting approximation is a Gaussian Markov Random Field (GMRF). This means a particular location’s spatial process depends only on that of its neighbors, not the entire map, markedly simplifying computation^24^. In our application of the SPDE approach, we parameterize the Matérn in terms of three parameters: $\sigma _s^2$ (spatial variance), $\rho _s$ (spatial range), and $\nu$ (shape, or smoothness).

In R-INLA, this discrete approximation is done using a set of non-overlapping triangles which together comprise a mesh over the study region. The basis functions are piecewise linear, equal to 1 on a given vertex and 0 on all other vertices. If one imagines elevating a single mesh vertex, a three-sided pyramid is formed, and the discrete approximation to the GP is a weighted combination of these pyramids. A projection matrix *A* projects from the mesh vertices to the *n* study locations (here, clusters).

Choice of the mesh dictates the resolution of the spatial effect, with a rule of thumb that features more than two triangles large are resolved well, while features smaller than a triangle will be biased in proportion with the triangle size^23^. Our meshes for Malawi, Uganda, and DRC are presented in Fig. 19 below. In all three countries, we set our mesh to have an inner and outer mesh, moving boundary effects away from the study area (i.e., country borders). We set the triangles to be smaller in the inner mesh (maximum edge length of 0.3$^{\circ }$) than the outer mesh (maximum edge length of 0.6$^{\circ }$), and the extension to be 1$^{\circ }$.

Predictors When the data are complex survey data, the sampling scheme may be accounted for by including design variables—district and urban/rural status in this case—in the regression model. As district is confounded by space, the spatial random effect (detailed above) accounts for this aspect of study design, and district was not included as a predictor in our models.

Predictors included urban/rural status (binary), protected areas (binary), elevation, and bodies of water (binary). While there are many definitions of urbanicity, as urban/rural status is a design variable, the desired definition for this predictor is the value that a given cluster would take had it been sampled by a household survey in a given year. As the data sources we used for livestock mapping largely used the most recent population and housing census as their sampling frame, for each country we generated a 1km$^2$ urban/rural surface for each sampling frame year. We accomplished this using logistic regression models, with population density from WorldPop^13^ and nighttime lights from the National Oceanographic and Atmospheric Administration’s Nighttime Lights Time Series^25^, as the predictors. For validation, we split the data into a 2/3 training and 1/3 test set, and evaluated model performance on the test set. We found these models performed very well for rural clusters (95% correct for Uganda, 96% for Malawi and DRC), however performance varied across countries for urban clusters (43% for Uganda, 86% for Malawi, 78% for DRC). As the vast majority of clusters in all three countries are rural, overall performance remained adequate.

Data on protected areas came from the World Database on Protected Areas^26,27^; while this database is updated monthly, there is no publicly-accessible archive. For bodies of water we used levels 1 (lakes $\ge$ 50km$^2$ and reservoirs $\ge$0.5km$^3$) and 2 (permanent open water bodies with a surface area of $\ge$0.1km$^2$) data from the Global Lakes and Wetlands Database^28^. Finally, we used 7.5-arc-second data—which have a root mean squared error of 26-30 meters—and median elevation from GMTED2010 for elevation^29^. Note, in contrast with GLW-3 these predictors were used to improve model fit, not to mask unsuitable pixels^10^.

Models We fit a three stage Bayesian hierarchical model as follows for the minimal model, fit separately for cattle and pigs, and separately for each country:

$$\begin{aligned}&Y_i|\mu _i, p_i\sim \text {ZIP}(\mu _i, p_i)\\&\quad \mu _{i}=exp\bigg (\alpha _0 + \varvec{\beta }_1 {{\textbf {x}}}_{i} + S({{\textbf {s}}}_i) + \omega _t + \epsilon _i + \gamma _k + \eta _t+ u({{\textbf {s}}}_i, t) + log(D_i)\bigg )\\&\quad S_i|S_j, j\in ne(i) \sim \text {GMRF}(\sigma _s^2, \rho _s)\\&\quad \omega _t|\omega _{t-1}, \omega _{t-2}, \omega _{t+1}, \omega _{t+2}\sim N\bigg (\frac{4}{6}(\omega _{t-1}+\omega _{t+1}) - \frac{1}{6}(\omega _{t-2}+\omega _{t+2}),\frac{\sigma _{\tau }^2}{6}\bigg )\\&\quad \epsilon _i|\sigma _{\epsilon }^2\sim _{iid} N(0, \sigma _{\epsilon }^2)\\&\quad \gamma _k|\sigma _{\gamma }^2\sim _{iid} N(0, \sigma _{\gamma }^2)\\&\quad \eta _t|\sigma _{\eta }^2\sim _{iid} N(0, \sigma _{\eta }^2) \end{aligned}$$

where *i* indexes cluster, $p_i$ is the hyperparameter that pertains to the model for the zeroes for cluster *i*, $Y_i$ is the number of livestock in cluster *i*, $D_i$ is the number of humans in cluster *i* (offset) $\alpha _0$ is the intercept $\varvec{\beta }$ is a vector of coefficients for the predictors ${{\textbf {x}}}_{i}$ is a vector of predictor variables for location *i* $S({{\textbf {s}}}_i)$ are the spatial random effects (error terms), assumed to follow a Markovian Gaussian random field (GMRF) with variance parameter $\sigma _s^2$ and range parameter $\rho _s$, and *ne*(*i*) are the neighbors of cluster *i*.

$\omega _t$ is a random walk 2 (RW2) random effect on time (years) $\epsilon _i$ is an unstructured cluster-level random effect (nugget) $\gamma _k$ is an unstructured random effect on survey $\eta _t$ is an unstructured random effect on time (years) $u({{\textbf {s}}}_i, t)$ is a space-time interaction which is approximated by an SPDE in space combined with an AR(1) process in time

We fit these models as type 1 zero-inflated Poisson (ZIP) models in R-INLA^30^, which combine a distribution for the proportion of zeros with the Poisson distribution. The type 1 likelihood is given as:

$$\begin{aligned} \text {Prob}(Y|\mu , p) = p \times 1_{y=0} + (1-p) \times \text {Poisson}(Y|\mu ) \end{aligned}$$

where the first part is the process that generates the zeros (i.e., clusters with no livestock; $1_{y_i=0}$ is an indicator that cluster *i* has 0 livestock), and the second part generates the livestock counts in cluster *i*. This likelihood is a mixture of structural zeros ($p_i$) and sample zeros ($1-p_i$)^31^.

For model selection, we fit a series of five models with equivalent random effects but varying fixed effects: intercept only (model 1); intercept and urban/rural (model 2); intercept urban/rural, and protected area (model 3); intercept, urban/rural, protected area, and bodies of water (model 4); and intercept, urban/rural, protected area, bodies of water, and elevation (model 5). We also fit a sixth model with an RW1 effect in time, rather than RW2, and all fixed effects included in model 5.

In DRC, due to the limited data availability, we fit models only from 2008-2015 (i.e., extrapolating 2 years in each direction from the input data), and we restricted random effects to the structured (SPDE) effect on space, the structured (RW1 or RW2, depending on the model) effect on time, and the unstructured (iid) effect on space (cluster). That is, the space-time interaction and unstructured random effects for survey and time were removed.

Priors As in Wakefield et al.^32^ for the spatial random effect we assigned a fixed shape $\nu =1$, and a “penalized complexity” (PC) prior^33,34^ for the spatial range $\rho _s$ and marginal standard deviation $\sigma _s$, such that $Pr(\rho _s< 0.3)=0.05$, and $Pr(\sigma _s>1)=0.05$. For the spatial range parameter, the selected priors can be interpreted as the 5% quantile corresponds to 0.3$^{\circ }$, which is approximately 4% and 9% of the extent of Malawi in the north-south and east-west directions, respectively, and approximately 5% and 1.5% of the extent of Uganda and DRC in each direction, respectively. This spatial range, as well as the mesh, were chosen to be adequately fine to allow for construction of a high-resolution raster map, but adequately coarse to account for jittering of cluster locations in the source data (a means to protect privacy) and to make the model computationally feasible. We used R-INLA’s default priors for the structured temporal effects (RW1 and RW2).

For the AR(1) process in time, again using the PC prior specification we set $Pr(\rho _t>0.5)=0.8$ as the prior. Finally, for the precision (inverse of variance) parameters for each of the independent and identically distributed (iid) random effects ($\sigma _{\epsilon }^{-2}, \sigma _{\gamma }^{-2}, \sigma _{\eta }^{-2}$), we specified the prior as $Pr(\sigma ^2>0.5)=0.01$.

Prediction on a regular grid After fitting our models, we projected livestock density on regular grid, generating 0.017$^{\circ }$ x 0.017$^{\circ }$ grid cells in each country (Fig. 20). This choice governed the resolution of our final raster maps.

We did not use any of the iid (unstructured) random effects for prediction as these were assumed to reflect measurement error. Thus, prediction was comprised of each model’s linear predictor, a vector of predictor values for each grid cell, the structured spatiotemporal random effects, and an appropriate projection matrix for each random effect. To produce estimates of uncertainty, we took 1000 draws from the posterior distribution of the structured random effects using the command inla.posterior.sample(), and present here the median and width of the posterior 95% credible interval over these 1000 estimates.

Because we did not use an offset in our predictions, we essentially “flattened” the distribution of the human population over the map: our final estimate is that of livestock counts in a grid cell with 1 human being, equivalent to the ratio of livestock to humans, which we are calling livestock “density”.

Model selection and cross-validation After fitting our models, we performed model selection via LOOCV for the cattle maps by survey, using mean squared error (MSE) as our measure of model performance. We defined hold-out sets by survey as we could most readily conceptualize the missing data as hypothetical surveys which were not conducted or samples not collected by a given survey, and we selected MSE due to its interpretability and its ability to capture bias-variance trade-off. Each fold left out a random 25% sample, distributed evenly among sampling strata defined by district and urban-rural status. We calculated MSE as:

$$\begin{aligned}\text {MSE}=\frac{1}{N} \sum \limits _{i=1}^N(y_{obs_i} - y_{pred_i})^2 \end{aligned}$$

where $y_{obs_i}$ is the observed density at location *i*, and $y_{pred_i}$ is the predicted density at location *i*.

External comparisons In addition to cross-validation, we compared our results with two external data sources: GLW-3 (dasymetric product)^35,36^ and national-level FAOSTAT total stock estimates^14^. By virtue of the dramatically different scales between our product and these products, these comparisons should be viewed as rough “plausibility checks” rather than formal validation.

As GLW-3 generated estimates of livestock counts for 2010 only, for this comparison we used WorldPop data as the denominator to derive density estimates^13^, and compared these with our 2010 estimates. We then regressed derived GLW density estimates against our estimates at the pixel-level, using linear regression, and reported coefficient size. For comparison with FAOSTAT total stock data, we used World Bank national (human) population estimates^15^ to convert stock counts to density estimates, and compared this with our estimated median density over all years, for each country.

#### South Sudan

Weighted estimates After reading in the data, we used the svyby() and svyratio() functions in the survey package in R^37^ to generate weighted estimates of county-level density, using sample weights contained in the IPUMS subset^22^ and the ratio estimator below, where *i* indexes household and *c* indexes county:

$$\begin{aligned} {\widehat{Y}}_{cHT}= & {} \sum \limits _{i=1}^{n_c} w_{ic}Y_{ic}\\ {\widehat{D}}_{cHT}= & {} \sum \limits _{i=1}^{n_c} w_{ic}D_{ic}\\ {\hat{\mu }}_c= & {} \frac{{\widehat{Y}}_{cHT}}{{\widehat{D}}_{cHT}} \end{aligned}$$

where $Y_{ic}$ is the number of livestock in household *i*, county *c*, and ${\widehat{Y}}_{cHT}$ is the Horwitz-Thompson estimate for the number of livestock in county *c*. $D_{ic}$ is the number of residents in household *i*, county *c*, and ${\widehat{D}}_{cHT}$ is the Horwitz-Thompson estimate for the (human) population in county *c*. $n_c$ is the total number of households in county *c*. $w_{ic}$ is the sampling weight for household *i* in county *c*, which is the inverse of sampling probability.

We also used the survey package to estimate design-based standard errors for livestock density. Adapted from Mercer et al.^38^ we then specified ${\hat{\theta }}_c = log({\hat{\mu }}_c)$, which, by the delta method, gives us the asymptotic sampling distribution:

$$\begin{aligned}{\hat{\theta }}_c|{\hat{\mu }}_c \sim N\bigg (log({\hat{\mu }}_c),\frac{{\widehat{var}}({\hat{\mu }}_c)}{{\hat{\mu }}_c^2}\bigg )\end{aligned}$$

Smoothing After generating the direct estimates (${\hat{\theta }}_c$) as detailed above, we next used spatial smoothing to stabilize the variance of ${\hat{\theta }}_c$ using a three-stage Bayesian hierarchical model, defined below (note random effects are defined on the transformed scale as in Mercer et al.^38^):

$$\begin{aligned}&{\hat{\theta }}_c|{\hat{\mu }}_c \sim N\bigg (log({\hat{\mu }}_c),\frac{{\widehat{var}}({\hat{\mu }}_c)}{{\hat{\mu }}_c^2}\bigg )\\&\quad {\hat{\theta }}_c = \beta _0 + \epsilon _c + S_c\\&\quad \epsilon _c|\sigma _{\epsilon }^2 \sim _{iid}N(0, \sigma _{\epsilon }^2)\\&\quad S_c|S_k, k \in ne(c) \sim N \bigg ({\overline{S}}_k, \frac{\sigma _s^2}{m_c}\bigg ) \end{aligned}$$

where

- $\epsilon _c$ are county-level iid (unstructured) random effects with variance $\sigma _{\epsilon }^2$
- $S_c$ are county-level structured random effects which follow the ICAR model with marginal variance $\sigma _s^2$
- *ne*(*c*) denotes neighbors (shared boundary) of county *c*
- $m_c$ is the number of neighbors of county *c*

and ICAR is the intrinsic conditional autoregressive model, which smooths each county’s random effect to that of its neighbors, with more smoothing performed for counties with fewer neighbors.

Priors As for the SPDE models, we used PC priors for the smoothing model, with $Pr(\sigma _{\epsilon })>1 = Pr(\sigma _s)>1 = 0.01$. This yields a posterior 95% credible interval for each random effect’s residual rate ratio of (0.36, 2.71)^33,34^.

Model selection As there was only one candidate model for South Sudan, model selection was not performed.

### Ethics approval

As this work relied solely on pre-existing, de-identified data, it does not constitute human or animal subjects research. Per the University of Washington Human Subjects Division, review and approval by the University of Washington Institutional Review Board is not needed (IRB ID: STUDY00004648).

## Data Availability

While existing data use agreements do not allow direct sharing of input data, all data were downloaded from publicly-available sources, detailed in Table 1, with links included in our References section. All analyses were performed in R, and all code are available in the GitHub repository linked in the Results section.
